# Using cameras to index waterfowl abundance in winter-flooded rice fields

**DOI:** 10.1016/j.mex.2020.101036

**Published:** 2020-08-19

**Authors:** Alexandra G. Firth, Beth H. Baker, Mary-Lynn Gibbs, John P. Brooks, Renotta Smith, Raymond Bruce Iglay, J. Brian Davis

**Affiliations:** a Department of Wildlife, Fisheries and Aquaculture, Mississippi State University, United States; b Genetics and Sustainable Agriculture Research Unit, USDA-ARS, United States

**Keywords:** Fecal matter quantification, Waterfowl, Rice fields, Bird surveys

## Abstract

Extensive wetland habitat loss across the continental United States has caused post-harvested rice fields to become an important surrogate wetland habitat for migratory waterfowl. Flooded rice fields used by waterfowl have the potential to provide agronomic benefits to soil. Increasing interest in the reciprocal relationship between birds and flooded rice fields has given rise to many studies that aim to quantify bird abundance. However, surveying large flocks of birds in open agricultural fields is challenging because traditional ground and aerial surveys can cause birds to flush or re-allocate spatially, thus biasing counts that are reflected in following management practice recommendations. To avoid this, we used camera surveys and an open-access image manipulation program to estimate 24-h bird use of rice fields. Indices of bird abundance from counts were used to estimate fecal matter input to rice fields. Camera surveys have the potential to limit biases seen in other methods because of their ability to capture bird use over a 24-h period over an entire season and the ability for multiple researchers to survey the same site.

• Surveying bird flocks by traditional ground or aerial surveys can bias bird abundance estimates.
• Camera surveys of waterfowl in rice fields were used to estimate bird abundance and fecal matter input.
• Camera surveys reflect static bird use over 24-h which can lower bias seen in traditional methods.

## Graphical abstract

**Figure:**
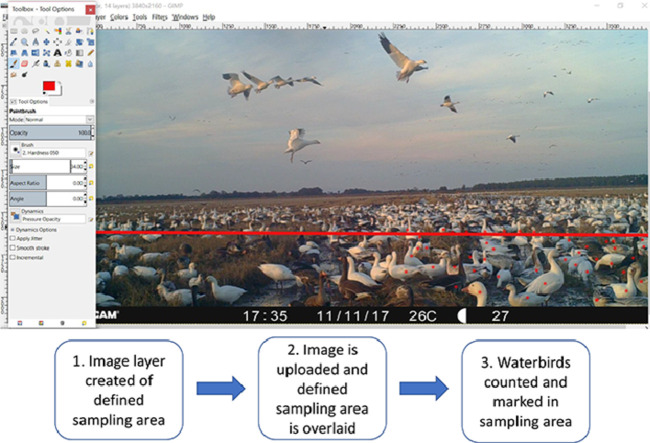

Specifications table

**Table:** 

Subject Area	Agriculture and Biological Sciences
More specific subject area	Bird use of flooded rice fields and management implications
Method name:	Using game cameras to standardize waterfowl surveys
Name and reference of original method	Wetlands Internation *Guidance on waterbird monitoring methodology: Field Protocol for waterbird counting*; 2010
Resource availability	https://www.gimp.org/

## Method details

### Justification for methodology

The Mississippi Alluvial Valley (MAV) once contained 10 million ha of bottomland hardwood forest, but extensive drainage and deforestation for flood control, agriculture, and other alternative uses has reduced these resources to approximately 2.8 million ha today, a 72% loss [1]. Amid extensive wetland habitat loss, post-harvested rice fields that are flooded have become important surrogate wetland habitats for waterbirds in the continental United States [2], [3], [4]. Management of flooded post-harvested rice fields is deemed important to conservation and a best management practice in the MAV by the Lower Mississippi Valley Joint Venture, a component of the North American Waterfowl Management Plan (NAWMP) [5]. In fact, rice fields of the MAV provide 11% of the total potential total food calories available to millions of waterfowl that winter each year in the MAV [5]. Additionally, flooded rice fields used by waterfowl have the potential to provide agronomic benefits to soil via fecal matter deposition and plant litter incorporation [6], [7], [8].

Several studies have used some type of survey to estimate abundance and diversity of avian guilds in flooded rice fields and to better understand the overall conservation value of these resources for birds [2,^3^,14]. Surveys may be conducted by ≥1 observer that walks or is concealed in a blind on the ground, or by aircraft flying at a low altitudes [9]. However, both methods can disturb birds, biasing results. Further bias in abundance estimates may result because concealed or surveyor's afoot have limited visibility. Alternatively, stratified random aerial surveys in winter can yield a precise estimate of regional duck abundances (10-15% CV; [10,11]. However, the significant challenge with using aerial surveys is that birds can be highly disturbed by approaching aircraft, flushing in multiple directions from the ground before reliable counts can be obtained. Thus, ground and aerial surveys for enumerating waterfowl, particularly geese, can be quite challenging and expensive. Alternative monitoring methods are needed.

Game cameras are common in wildlife research, especially for mammal surveys or bird nest monitoring [12]. Game cameras have received little use for surveys of flocking birds, presumably because of their limited field of view. However, considering bias associated with traditional waterfowl surveys, using cameras in an open area, such as rice fields, could help mitigate inaccuracies and reduce bias. Herein, we present methodology for using game cameras and a free image manipulation program, to index waterfowl abundance in winter flooded rice fields. We wanted to quantify waterfowl abundances, as part of a larger study to estimate fecal matter additions to rice fields by waterfowl.

### Camera survey methodology

Bird surveys were conducted on two rice production sites: (1) a Low-external-input-sustainable agriculture (LEISA) site, which has been flooding select fields for bird use since 2009, and (2) conventional, which had never flooded fields for bird use. Each site applied a flooded or non-flooded treatment regime for field selection. Treatments included: (1) LEISA flooded (LF; long-term flooding management, no-till fields), (2) LEISA non-flooded (LN; long-term non-flooded, no till), (3) conventional flooded (CF; first year flooded, tilled) and (4) conventional non-flooded (CN; never flooded, tilled).

Initial bi-weekly counts of waterfowl were conducted from a concealed blind within fields during winter 2016-2017. One person identified and counted waterfowl species in a field that was either bounded by small levees or by counting out to the natural field edge. However, because of the openness of rice fields and little opportunity to move from field to field undetected during surveys, we realized the close proximity of human observers and birds caused them to flush or un-naturally re-distribute, creating biased counts. Given these challenges, we ceased traditional ground surveys and began camera surveys.

We initiated camera surveys in winter 2017-2018, using no-glow infrared camera traps (Stealth Cam® G42NG). In each field <20 ha, we placed one camera 15 m interior to the center of the southern field edge. Each camera was mounted to a T-post, faced north from 91 cm above ground, and was secured with plastic coated wire at a 90° angle. For fields ≥ 20 ha, we placed a second camera, centered and 15 m interior to the northern field edge, and facing south. We had one LN field > 40 ha in which we installed three cameras (i.e., one camera along the southern field edge and two cameras in the northern corners facing southeast and southwest, respectively). A metal stake was placed 30.4 m directly in front of each camera as a distance reference point to define the sampling area during image evaluations. Therefore, each camera had a 242.32 m² sampling area directly in front of each camera, per the reference stake and standardized camera placement. Camera placement in this manner corroborated with the camera's maximum clarity of vision range as recommended by the manufacturer.

Cameras were programmed to capture one picture hourly from November 3, 2017 through March 15, 2018. Camera data cards were retrieved and replaced monthly. Camera theft (*n* = 3 cameras in January 2018), failure (*n* = 2 cameras in December 2017), and obstruction from falling over (*n* = 2 cameras in January 2018) resulted in missing pictures for those periods. Thus, pictures from these fields were only available for part of the sampling period.

Images from camera traps were downloaded and opened in GNU Image Manipulation Program (GIMP) to index bird abundance [13]. An image layer was created that clearly defined the entire 242.32m² sampling area captured by the camera, using the marker stake as reference. The image layer was overlaid on each discernible photo, and waterfowl were counted and recorded for each hour in each field. Non-waterfowl birds accounted for less than 10% of the observed species and therefore excluded from analysis. To ensure count accuracy, individual birds were marked with a red dot to avoid re-counting. Over the course of the season, reference stakes from many cameras disappeared. By creating an image layer for each camera at the beginning of the season and reusing it for each count, we had consistent and well-defined sampling area for a counting birds.

We addressed potential count bias of waterfowl species identification and abundances using a double observer approach. Briefly, the lead researcher first interpreted all of the photographs and logged species and abundances. Thereafter, a subset of photographs was randomly selected (*n* = 30) and counted independently by the lead and second observer. Independent counts were then compared using a one-way analysis of variance (ANOVA). We found no difference between observer counts *(F_2,87_* =0.001, *P*=0.999; i.e., no observer bias).

We used five steps to estimate bird counts in respective fields: (1) Geese and ducks were counted separately in the defined area (242.232 m²) and averaged for every day (24-h period) pictures were available, (2) the average number of geese and ducks per day in 1 m²/field was calculated, (3) converted to geese and ducks day per ha, (4) averaged among cameras in the same field (geese and duck/day/ha), and (5) then averaged across the season based on the number of active camera days per field. To ultimately determine fecal inputs, we used average number of waterfowl/day/ha based on the number of active cameras.

### Application of camera surveys: fecal estimates

Total fecal inputs by waterfowl to fields were based on dry weights of bird droppings per day. We used published values from existing literature because we lacked the resources to collect fecal samples in the field and analyze them in the laboratory. Although white-fronted geese (*Anser albifrons*) and snow geese (*Chen caerulescens*) were the common species using our fields, published estimates were more readily available for Canada geese (*Branta canadensis*) fecal matter (Terres, 1980). However, all of these geese consume agricultural grains and green browse, which occurred in our study fields. So we deemed Canada geese representative of our species. Moreover, we used the published estimates for mallards (*Anas platyrhynchos*; Sanderson and Anderson, 1978) to represent ducks in our study. Mallards commonly use rice fields in Mississippi. Thus, these species provide a reasonable average dropping rate of their respective bird guilds [14], [15], [16], [17]. From the published studies, average dropping rates for geese and mallards were 81.6 and 27.0 g per day, respectively (Sanderson and Anderson, 1978; Terres, 1980). We applied these values to estimate average total dry grams of fecal input per field ha⁻¹ day^−1^ during winter. Fecal inputs by geese and ducks per ha⁻¹ day^−1^ were summed for an overall estimate of fecal matter input to fields.

### Comparing waterbird estimations and advantages of camera surveys

When comparing mean abundances of geese in camera surveys used in this study to ground survey estimates in published literature, cameras yielded greater abundances of geese and ducks in most field types (Table 1) [18,19].

**Table 1:** Comparison of mean waterbird abundances in camera surveys and ground surveys.

Camera Surveys (Firth et. al 2020)			Ground surveys (Sesser et. al 2016)			Ground Surveys (Marty 2013)	
Field type	Geese/ ha/day ($\bar{x}$)	Duck/ ha/day ($\bar{x}$)	Field type	Geese/ ha/day ($\bar{x}$)	Duck/ ha/day ($\bar{x}$)	Field type	Waterbird/ ha/day ($\bar{x}$)
Long-term flooded, no till (LF)	15.8	6.1	Flooded, non-baled	5	7	Flooded, non-disked	7.35
Long-term non-flooded, no till (LN)	7.2	2.6	Non-flooded, non-baled	3	0	Non-flooded, non-disked	0.07
First year flooded, tilled (CF)	5.94	7.7	Flooded, baled	3	3	Flooded, disked	3.35
Non-flooded, tilled (CN)	2.12	3.2	Non-flooded, baled	5	0	Non-flooded, disked	0.06

Field types are aligned based off management similarity (flooding regime and post-harvest management). Only means are reported, see original article for full descriptive statistics [8,^18^,19]. Sesser et al. and Marty are approximations of reported values.

A primary benefit of camera surveys is that bird abundances are captured over a full 24-h cycle, whereas ground and aerial surveys are conducted only during daylight. Capturing nocturnal waterfowl abundances has been particularly elusive to researchers but is an important metric, such as in relation to food depletion, or in this case, nutrient deposition in fields. The advantage of the 24-h camera survey also relates to the basic biology and behavior of waterfowl. For instance, ducks mostly forage in flooded fields of the MAV. Abundances of ducks in a field diurnally and nocturnally are often divergent. Although it is possible to have a rice field contain more ducks diurnally than nocturnally, it often is the reverse. Ducks may be feeding or loafing elsewhere during the day, then return to flooded habitats (e.g., rice fields) and roost by the thousands [16,20]. Although ducks may not always be foraging in rice fields nocturnally, they still defecate in the water. The challenge of obtaining estimates of goose abundances is even worse. That is, geese will forage among dry, muddy, and flooded fields diurnally but retreat to flooded habitats exclusively at night for roosting. Hence, not accounting for nocturnal goose abundance in rice fields, which on any given night may be in the tens-of-thousands depending on several factors, severely under-estimates, in our case, fecal matter estimates. Photographs also allow for more accuracy in species identification and opportunity for multiple researchers to survey the same site and point in time. This further reduces biased results and develops a long-term digital archive.

## Declaration of Competing Interest

The authors confirm that there are no conflicts of interest.
